# Recent advances in carbon-based materials derived from diverse green biowaste for sensing applications: a comprehensive overview from the perspective of synthesis method and application

**DOI:** 10.1039/d4ra07693a

**Published:** 2024-12-17

**Authors:** Saade Abdalkareem Jasim, Nikunj Rachchh, Harikumar Pallathadka, R. Sanjeevi, Dmitry Olegovich Bokov, Shoira Formanova Bobonazarovna, Hijran Sanaan Jabbar, Shriya Mahajan, Yasser Fakri Mustafa, Merwa Alhadrawi

**Affiliations:** a Medical Laboratory Techniques Department, College of Health and Medical Technology, University of Al-maarif Anbar Iraq; b Marwadi University Research Center, Department of Mechanical Engineering, Faculty of Engineering & Technology, Marwadi University Rajkot-360003 Gujarat India; c Manipur International University Imphal Manipur India pallathadkaharikumar@gmail.com; d NIMS School of Allied Sciences and Technology, NIMS University Rajasthan Jaipur 303121 India; e Institute of Pharmacy Named After A. P. Nelyubin, Sechenov First Moscow State Medical University 8 Trubetskaya St., Bldg. 2 Moscow 119991 Russian Federation; f Laboratory of Food Chemistry, Federal Research Center of Nutrition, Biotechnology and Food Safety 2/14 Ustyinsky pr. Moscow 109240 Russian Federation; g Department of Chemistry and Its Teaching Methods, Tashkent State Pedagogical University Tashkent Uzbekistan; h Department of Chemistry, College of Science, Salahaddin University-Erbil Kurdistan Region Iraq; i Centre of Research Impact and Outcome, Chitkara University Rajpura-140417 Punjab India; j Department of Pharmaceutical Chemistry, College of Pharmacy, University of Mosul Mosul-41001 Iraq; k Department of Refrigeration and Air Conditioning Techniques, College of Technical Engineering, The Islamic University Najaf Iraq; l Research Center, Knowledge University Kirkuk Road 44001 Erbil Iraq; m Department of Refrigeration and Air Conditioning Techniques, College of Technical Engineering, The Islamic University of Al Diwaniyah Al Diwaniyah Iraq; n Department of Refrigeration and Air Conditioning Techniques, College of Technical Engineering, The Islamic University of Babylon Babylon Iraq

## Abstract

The rapid increase in global waste, driven by population growth, has raised significant environmental concerns. Among different types of wastes, green biowastes (BWs) containing organic matter have attracted considerable attention. The conversion of BW, particularly from herbaceous and animal sources, to carbon-based materials (CBMs) introduces a suitable platform for waste management and resource recovery. Furthermore, this strategy creates valuable materials from low-value waste for various applications, sensing included. The abundance of these wastes provides a sustainable and affordable raw material and enhances the feasibility of fabricating these materials. Generally, the presence of carbon in their structure can present an accessible resource for producing different carbon materials, especially carbon dots (CDs), carbon quantum dots (CQDs), and graphene quantum dots (GQDs). The performance of these CBMs has been enhanced by optimizing synthesis processes, incorporating functional groups, and integrating various materials. The synthesized CBMs possess desirable features, such as biocompatibility, excellent physical, chemical, and electrical conductivity. These materials have been used in different sensors such as electrochemical (EC) and optical sensors for presenting high performance sensing probes with several benefits such as real-time monitoring, rapid detection, and high sensitivity. The first section of this review is dedicated to the preparation of CBMs, derived from green BWs, by different synthesized methods for use in different fields including biomedical application, food safety, and environmental monitoring. In addition, the challenges, limitations, and future directions in the development of these CBMs were completely discussed to improve their performance.

## Abstract 2

Carbon-based materials derived from green biowaste for biomedical application, environmental monitoring, and food safety analysis.

## Graphical abstract

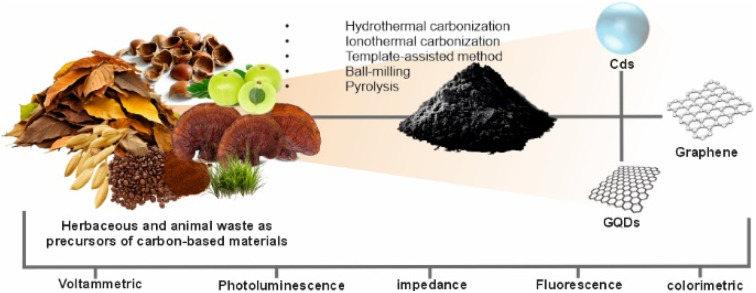

## 1. Introduction

The rapid increase in human population and urbanization has driven substantial expansion across various industries, resulting in different kinds of waste with varied challenges and far-reaching concerns for public health, the environment, and sustainable development. In other words, the production of a significant amount of waste, approximately 8 billion tons, poses a considerable obstacle for both the scientific community and society.^1,2^

While microorganisms can break down various biowastes (BWs), the detrimental consequences of their disposal are undeniable.^3,4^ Over the last few decades, numerous studies have investigated the importance of different non-bio solid waste, such as plastic, agricultural, electronic, and industrial wastes. However, the importance of BWs and their conversion methods has received attention to some extent. Generally, BW is considered less important compared to non-BW due to its low toxicity.^5–7^ On the other hand, it has a negative consequence on the environment by generating gases when burning, which contain carbon monoxide, nitrogen oxide, and sulfur dioxide, released into the atmosphere. Moreover, the increasing amount of animal waste has a detrimental effect on aquatic life owing to the reduction of oxygen levels in the water and the presence of pathogens in animal waste poses a significant threat to human health.^8^

According to the crucial need for innovative and comprehensive solutions to address the global waste issue, numerous scholars are paying special attention to transforming BWs into valuable resources. Over the last decade, several studies have been established for the analysis of BW.^9^ In general, BW consists of three main groups, including hemicellulose, lignin, and cellulose. On the other hand, there are significant variations in the chemical composition of these wastes owing to the source and treatment techniques.^10^ The basic framework of these wastes consists of carbon (C), oxygen (O), hydrogen (H), and also small concentrations of nitrogen (N) and sulfur (S) elements that connect to form polymeric structures. Furthermore, trace amounts of manganese (Mn), aluminum (Al), calcium (Ca), sodium (Na), silicon (Si), magnesium (Mg), chlorine (Cl), iron (Fe), potassium (K), and phosphorus (P) have been identified in these wastes.^11,12^ The natural source BWs includes three important categories including herbaceous, woody, and animal waste. The chemical compositions of the foremost groups of the BW are summarized in Table 1.

**Table 1:** Chemical composition of important green BWs^13^

Waste group	C %	H %	O %	S %	N %	Fixed carbon %	Moisture %	Ash %
Herbaceous waste	42–58	3–9	34–49	<1	1–3	9–35	4–48	1–19
Woody waste	49–57	5–10	32–45	<1	1	6–25	5–63	1–8
Animal waste	57–61	7–8	21–25	<1	6–12	12–13	3–9	23–34

According to high rate of carbon in the waste of different herbaceous and animal sources, these materials have been widely exploited as excellent natural carbon precursors for creating waste-derived carbon-based materials (CBMs). These materials consist of diverse and attractive family, including affordable graphite and expensive diamond, and are at the forefront of sensing applications. The discovery of new types of carbon-based composites such as fullerene, carbon nanotubes (CNTs), and graphene in 1985, 1991, and 2004 revolutionized technology and material science.

**Fig. 1:**
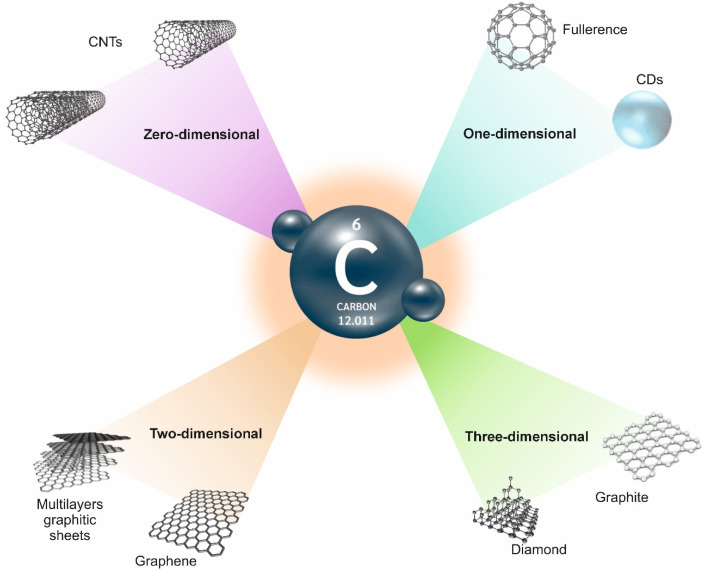
Different forms of carbon allotropes.

Up to now, different carbon-based composites, including fullerene (zero-dimensional) carbon nanofibers, carbon black, carbon nitrides, carbon dots (CDs), CNTs (one-dimensional), graphene sheet (two-dimensional), graphite (three-dimensional), and graphene quantum dots (GQDs) are not isolated from each other, however in a close relationship.^14,15^ For instance, graphene has been exploited as a building block of all other graphitic carbon allotropes with different dimensionality. Considering the various possible properties, intriguing electrical, optical and mechanical features of CBMs have ensured new directions in various sensors. In detail, chemical stability, high electrical conductivity, strong mechanical strength, biocompatibility, easy functionalization, and biodegradability are important advantages of these materials.^16^ Furthermore, modification of these CBMs with different nanomaterials improved their performance. The common forms of carbon allotropes are demonstrated in Fig. 1.

The inherent characteristics confer interesting features to the prepared CBMs, which are derived from BW, to apply in diverse sensing approaches including electrochemical (EC),^17^ optical,^18^ and piezoelectric sensors^19^ for analysis of various targets. Recently, waste-derived CBMs have attracted considerable attention owing to the excellent EC and optical activities for several redox reactions, additionally, affordability, and easy modification.

Over the past few years, numerous researchers have reviewed the application of various waste sources for creating materials in sensing due to affordability, renewability, and sustainability. For example, Bosu *et al.*,^20^ reviewed the sensing approaches based on green CDs for detection of metallic contaminants in the environment. Furthermore, Malode and co-workers,^21^ highlighted a application of carbon materials for sensor applications. In 2022, Blessy Rebecca and colleagues,^22^ reported a review study showing the potential of green graphene-based materials for biomedical applications. However, there is no comprehensive review for revealing the application of BW-derived CBMs in biomedical application, food safety, and environmental monitoring.

In this review, the implementation of the obtained CBMs in sensors is considered a promising candidate for introducing high performance probes. In addition, different synthesis methods of carbon-based nanomaterials are classified in this review and several analytical parameters using different CBMs were completely discussed.

## 2. Herbaceous and animal waste as precursors of carbon-based materials

BW contains organic materials that can be decomposed, primarily by microorganisms, into small compounds.^23^ Herbaceous and animal waste, two common types of green BW, have shown promising results in producing CBMs. Various types of herbaceous waste and plant byproducts, including fruit-derived waste, leaf-derived waste, grain-derived waste, and seed-derived waste, have been obtained from agricultural activities and are particularly promising precursors of CBMs.^24,25^ Herbaceous waste and its derivatives are easy to process and affordable, introducing a suitable source of CBMs.^26^ Coffee waste, for instance, is one of the accessible and affordable plant-derived food waste, that contains high levels of carbon. 10.3 million metric tons of coffee beans were produced in 2018, according to the International Coffee Organization (ICO).^27–29^ Despite the various positive effects of coffee on human health and the world economy, the waste produced from coffee consumption has diverse negative consequences on the environment and economy.^30^ Particularly, in terms of negative economic consequences, the most recent data available from the ICO, the US purchased 27.7 million 60 kilogram bags of coffee.^31^ The cost of coffee waste disposal is estimated at $70.2 million, assuming landfills are the primary destination.^32,33^ Along with economic drawbacks, the negative environmental impacts of coffee waste are undeniable. The production of toxic chemicals from coffee processing industries, such as tannin, caffeine, and polyphenols, can contaminate water.^34^ These chemicals may hinder the germination and root elongation of specific plants and create anaerobic circumstances which harmful to aquatic habitats.^35,36^ Furthermore, coffee waste in landfills emits greenhouse gases such as methane and carbon dioxide, contributing to global warming.

Another important BW is animal waste which has the potential to become a valuable resource. In general, this type of waste can be obtained from dairy products and chicken byproducts.^37^ Every year, millions of tons of waste are produced in livestock operations (such as cattle, poultry, and swine) and seafood. For example, in 2006, 6 million tons of shrimp were produced globally and only 60% were consumed, producing 2.3 million tons of inedible waste.^38,39^ These wastes are resources to regain natural elements chondroitin sulphate and they can be considered as carbon precursors. Along with seafood, the high demand for meat and chicken leads to developed meat and poultry industries.^40^ The advancement of these industries has a direct relationship with producing inedible parts such as feathers, bones, tendons, and skins. Converting animal waste can prevent the growth of pathogen bacteria.

Several drawbacks of BW on the environment coerce scholars into developing efficient synthesizing methods of CBMs from herbaceous and animal waste. In the next section, the synthesized method and its advantages and disadvantages are completely discussed.

## 3. Synthesis methods

A variety of synthesis methods have been developed for CBMs derived from BW due to the different requirements and wide applications of CBMs. Indeed, the important role of synthesis technique and associated factors, such as temperature, pH, pressure, solvent, residence time, gas atmosphere, and substrate concentration, is incredibly significant in determining the activity and the structure of synthesized materials. The desirable properties of BW-derived CBMs for sensing application are achieved by controlling synthesis parameters and using appropriate methods.^41,42^ Among different techniques, hydrothermal carbonization, ionothermal carbonization, pyrolysis, template-assisted method, and ball-milling approaches have attracted considerable attention for converting BW into high-value materials.

### 3.1. Pyrolysis

Pyrolysis is a widely employed method for transforming organic materials into CBMs. This process involves decomposition and carbonization of precursor material, at high temperatures and in the absence of oxygen to produce biochar, bio-oil, and syngas. During pyrolysis, biochar was used as a precursor for CBMs. The optimal parameters of the process, such as composition of the organic precursor, heat-treatment temperature, influence of catalysts, and the presence of post-pyrolysis steps during heat treatment, can impact the morphology and physicochemical properties of resulting CBMs.^43,44^

### 3.2. Hydrothermal carbonization

Hydrothermal, as a thermochemical method, mimics the natural carbonization method for turning herbaceous and animal waste into CBMs at a fast rate. This technique is operated according to the wet biomass under high pressure and temperature for the decomposition and carbonization of the biomass.^45,46^ In the hydrothermal carbonization process, complex organic compounds are experienced thermal degradation and polymerization, leading the production of carbon-rich material.^47^ Under optimal conditions of hydrothermal carbonization method, such as preparation of the waste, loading into a reactor, and addition of water, temperature, and pressure, the hydrochar is synthesized. Under specific conditions, the formation of nanoscale carbon structures is also possible. According to the green concept ability of this method, which operates without the need energy intensive drying and minimum emission of methane, it can introduce green precursor, sustainable, non-hazardous, and quality-controllable CBMs.^48–50^

### 3.3. Ionothermal carbonization

In this approach, ionic liquids have been exploited as critical compounds due to their specific properties including low solvent volatility, thermal stability, and high solubility with the waste. The application of these liquids for template and solvent eliminates the competition between template-catalyst and solvent-catalyst. The obtained CBMs from the method enjoy desirable benefits, including excellent heteroatom doping, high surface area, well-structure, and high carbonization yields. These characteristics make ionothermal carbonization a high potential method for synthesizing ideal CBMs.^51^

### 3.4. Ball-milling

Ball-milling has become an efficient method for producing CBMs from BW. This process uses mechanical energy to break chemical bonds and fracture particles without the need for hazardous wet chemicals.^52^ The rotating container containing the balls employed mechanical forces on the material, resulting in the fragmentation and size reduction of the particles. Consequently, this process facilitates the production of a uniform and fine powder, suitable for subsequent processing. The ability of technique to exfoliate graphitic layers and induce surface functionalization of graphene nanoplatelets has been utilized to convert the waste into CBMs. Furthermore, this approach can enable researchers to control the morphology and particle size of CBMs.^53,54^

### 3.5. Template-assisted method

In order to craft porous carbonaceous materials with accurate morphology and structure, the template-assisted method can be considered a powerful method. Indeed, the application of template guides in growth CBMs creates hollow microporous structures, enjoying high surface area and a multitude of active sites, both of which improve physicochemical features. The disadvantages and advantages of different synthesis methods of CBMs from BW are summarized in Table 2.

**Table 2:** Comparison of different techniques of synthesizing CBMs derived from BW

Technique	Benefits	Drawback	Possibility of green synthesis	Ref.
Pyrolysis	Adjustable features	Low performance of production, air pollution, complexity of mechanism, and limited diversity of functional groups	High	55
	User friendly			
Hydrothermal carbonization	Less toxicity	Production of large size CBMs and require sealed vessels	High	56
	Adjustable morphology			
Ionothermal carbonization	Operated without high temperatures and sealed vessels	High cost	Low	57
Ball-milling	Flexible mechanisms, scalability, and operated without need harsh chemicals	Energy-intensive, require optimization	Low	58
Template-assisted method	Highly porous material	Incompletely removing template, low stability, and high cost	Low	59

## 4. Application of carbon-based materials in detection

Advancements in synthesis methods of CBMs derived from green BW are the key to the future success of carbon chemistry and its suitability for environmental fields. The good electron transfer capabilities and excellent surface characteristics of herbaceous and animal waste-derived CBMs presented them as exceptional candidates for sensing applications. Furthermore, distinctive properties, such as biocompatibility, affordability, accessibility, stability, and high surface area, are other reasons for using these CBMs in the structure of optical and the EC sensors. There have been several attempts to use BW-derived CBMs in the structure of EC and optical sensors for biomedical application, food safety, and environmental monitoring. The advantages of these CBMs on the surface of EC electrode provided an excellent surface for incorporation with different targets. Furthermore, these CBMs can play an important role as optical probes in optical sensors. Here, we focused on the application of these CBMs in the structure of sensors for detection of numerous targets.

### 4.1. Biomedical application

**Fig. 2:**
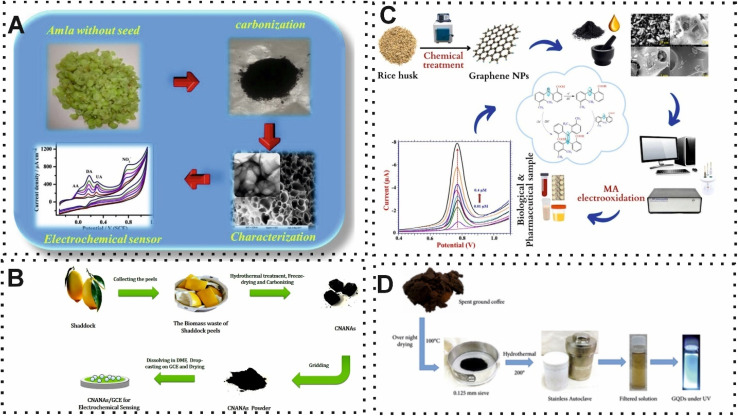
(A) Representation of using hierarchical porous carbon which derived from waste of amla for quantification of multiple biomolecules. Reproduced with permission from ref. 63. Copyright Elsevier Science, 2019. (B) Schematic of obtained and modification of EC electrode by hierarchical carbon nanoballs-based aerogel for inorganic and organic molecules quantification. Reproduced with permission from ref. 64. Copyright Elsevier Science, 2018. (C) Schematic illustration of EC sensor based on the BW-derived graphene for mefenamic acid quantification. Reproduced with permission from ref. 65. Copyright Elsevier Science, 2023. (D) Illustration of synthesis and preparation of B-GQDs for using in photoluminescence sensor in terms of glucose quantification.^66^

From the earliest recorded human civilizations to the modern era, diseases have been considered an important threat to human existence, causing widespread suffering and death. Biomedical knowledge and technology have directly or indirectly positive impacts on many diseases. Among them, the importance of analytical approaches in biomedicine is highlighted in the detection of abnormalities that may cause multiple disorders.^60,61^ Various research studies have been conducted to explore the potential of synthesizing CBMs, including hierarchical porous carbon, graphene, and CDs, from BW in the structure of biosensors for detection of biologically significant compounds ascorbic acid, dopamine, γ-aminobutyric acid, glucose, mefenamic acid, neurotransmitter, as well as for the diagnosis of Parkinson's disease, and cancer diagnosis. Most recently, Ostertag and co-workers,^62^ reported a novel and green EC sensor that exploited synthesized hierarchical porous carbon structures from waste coffee grounds for neurotransmitter quantification. In this protocol, the synthesis method was optimized for the porous carbon *via* chemical activation to achieve uniformity and EC determination. The desired surface characteristics and porous structure were achieved by controlling type and concentration of activating agents and temperature during the activation. The presence of a well-distributed pore and high surface roughness structure on the surface of EC electrode provided a brilliant situation for detection of neurotransmitter. However, the authors do not provide explicit details regarding the limit of detection (LOD) for detection of neurotransmitters. Similarly, Sudha and colleagues,^63^ used waste amla-derived hierarchical porous carbon in the structure of the EC sensor for simultaneous detection of ascorbic acid, dopamine, uric acid, and nitrite (Fig. 2A). The modification of EC electrode with the hierarchical porous carbon demonstrated an exceptional EC oxidation toward multiple biomolecules due to the high electrical conductivity and surface area of synthesis hierarchical porous carbon. The developed EC device detected ascorbic acid, dopamine, uric acid, and nitrite with a detection limit of 13.7 μM, 3.2 μM, 1.1 μM, and 3.3 μM, respectively. In 2018, another hierarchical carbon nanoballs-based aerogel for determination of inorganic and organic molecules was fabricated by Sha and co-workers.^64^ As shown in Fig. 2B, the surface of EC electrode was modified with the shaddock (*Citrus maxima*) peels-derived carbon nanoballs aggregation networks-based aerogels (CNANAs). Although the synthesized BW CBMs had a high surface area (446.39 m^2^ g^−1^), the detection limit of reported EC sensing approach was at the micro level.

Another important BW-derived carbon-based material is graphene, which is characterized by a hexagonal lattice structure that constructs one-atom-thick carbon sheets. This carbon-based material has garnered substantial attention due to the low synthesis cost compared to other CBMs. One instance of this BW-derived carbon-based nanomaterials for mefenamic acid detection was developed by Malode *et al.*^65^ In this work, the obtained high surface area and conductive carbon-based nanomaterials from rice husk, which activated with chemical producer, were used to decorate the surface of carbon paste electrode (Fig. 2C). In the presence of the target, the measurement of voltammetric response provided a LOD and linear range of 2.13 nM and 1.0 × 10^−8^ to 4.0 × 10^−4^ M, respectively. The reported EC sensor demonstrated an excellent recovery rate in real samples such as blood serum, human urine, breast milk samples, and pharmaceutical tablets due to the operation without excipient interference from co-existing molecules. In another study, boron was used in the structure of graphene for an efficient photoluminescence sensor of glucose.^66^ The introduction of boron into the graphene domain facilitates the application of GQDs which was achieved from coffee waste through a simple one-step hydrothermal process (Fig. 2D). Elaborately, according to the author's claim, the performance of boron-GQDs (B-GQDs) is approximately 26% better than GQDs. Under normal circumstances, the photoluminescence intensity has an indirect relationship with the concertation of glucose. Along with the exceptional optical features of GQDs, these CBMs derived from coffee waste can connect with numerous molecules through π–π or covalent bonds.

**Fig. 3:**
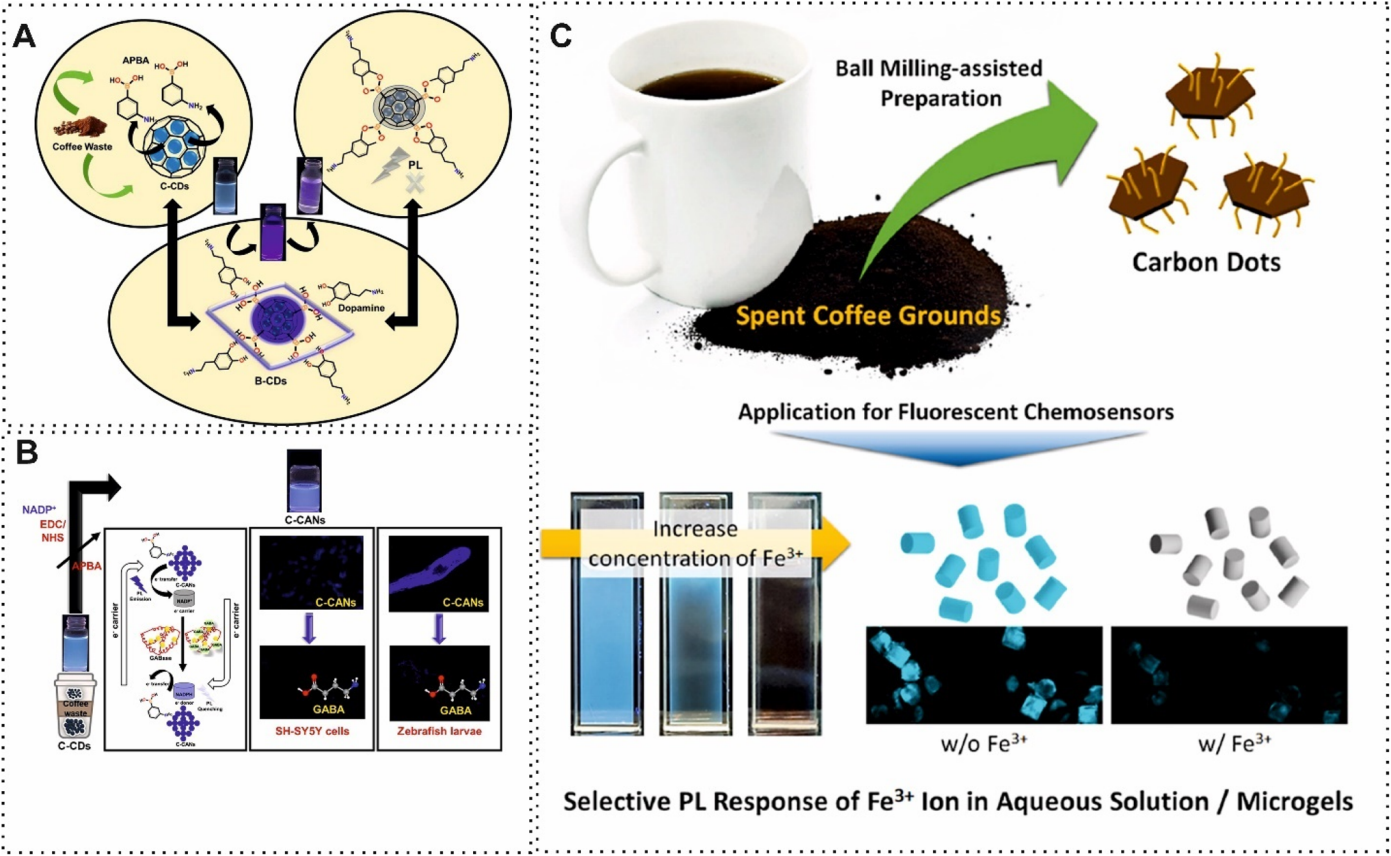
(A) Schematic of fluorescence biosensor based on extracted B-CDs from waste coffee for dopamine detection. Reproduced with permission from ref. 72. Copyright Elsevier Science, 2023. (B) Representation of synthesis and functionalized CDs for γ-aminobutyric detection. Reproduced with permission from ref. 73 Copyright Elsevier Science, 2023. (C). Schematic illustration of carboxylic acid-functionalized CDs for detection of Fe^3+^. Reproduced with permission from ref. 74 Copyright Elsevier Science, 2023.

Another commonly obtained materials from BW are CDs, which have a spherical shape and an average size of 10 nm. This carbon material exhibits good dispersibility, high photostability, biocompatibility, and strong fluorescence properties.^67,68^ One of the excellent examples of using tender coconut waste for determination of Ethionamide based on CDs in the structure of fluorescence was constructed by Gunjal and co-workers.^69^ The quenching of fluorescence of CDs with different concentrations of Ethionamide provided a detection limit of 0.33 μg mL^−1^. Similarly, Won *et al.*,^70^ presented another fluorescence sensor for detection of Fe^3+^ and ascorbic acid. In this study, an eco-friendly and affordable synthesis method and purification system was designed by using filtration, evaporation, and hydrothermal treatment. In terms of detection of Fe^3+^ and ascorbic acid, the recovery of optical signal with the addition of the targets was considered the principle of detection. In detail, Fe^3+^ preferred to binding oxygen-containing functional groups due to hard acid–hard base interactions which led to the transfer of electrons from the conduction band of the CDs to the unfilled d-orbitals of Fe^3+^. Hence, Fe^3+^ was detected by the turn-off quenching process. However, in the presence of ascorbic acid, the Fe^3+^ converted to Fe^2+^ which did not bind with oxygen-containing functional groups, leading turn-on quenching process. This reported sensing approach based on CDs was able to detect Fe^3+^ in the range of micromolar detection limit, which is not highly sensitive. In this regard, some scholars tried to improve the performance of obtained CDs from BW by using functionalizing agents and different atoms. For example, the modified BW-derived CBMs possess exceptional dispersion stability in a hydrophilic solvent. Recently, Wang and colleagues,^71^ applied seeds of green pepper-derived fluorescent N, S, P co-doped CDs in a fluorescence sensor for quantification of Fe^3+^. The optical properties, biocompatibility and photo-stability of the CD were improved by adding N, S, P. The presence of a large number of phosphate groups on the surface facilitated the Fe^3+^ bind through formation of Fe–O–P bonds. This phenomenon improved quenching of fluorescence signal. Another outstanding example of using functionalized groups in BW-derived CBMs was developed by Sangubotla and co-workers.^72^ In this protocol, they fabricated a novel fluorescence sensor based on B-CDs for dopamine detection (Fig. 3A). For this purpose, the surface of CDs was decorated with 3-aminophenylboronic acid through the ethyl(dimethylaminopropyl)carbodiimide and *N*-hydroxysuccinimide (EDC/NHS) reaction. Under optimal conditions, B-CDs demonstrated fluorescence quenching against dopamine concentrations in the range 0 to 30 μM with a LOD of 4.25 nM. In 2023, Sangubotla and colleagues,^73^ constructed a novel fluorescence biosensor using modified CDs, derived from coffee waste, with 3-aminophenyl boronic acid and nicotinamide adenine dinucleotide phosphate (NADP^+^) for γ-aminobutyric acid quantification. The NADP^+^ could improve sensitivity due to the redox cycling amplification role. As shown in Fig. 3B, boronic acids were used in redox cycling processes which led to signal amplification in biosensing. In addition, NADPH, a redox cofactor, can take part in these cycle processes. Therefore, thanks to the functionalized CDs, the reported fluorescence was able to detect γ-aminobutyric acid in the nanomolar range. The performance of modification of boronic acid on the surface of BW-derived CBMs was improved by using ethyleneimine (PEI) which was grafted onto the surface of the CBMs through covalently binding. Subsequently, boronic acid is immobilized *via* an amine aldehyde condensation reaction. The improvement of decoration surface of waste tea-derived CBMs by PEI was exploited for recognition of glycoprotein.^75^ Although the B-CDs have more advantages for chemical reactivity and functionalization, the concept of doped boron atoms in the structure of BW-derived CBMs has improved the conductivity of these materials. One of the excellent examples of this concept was achieved by derived B-CDs from Chinese herbal for towards Fe^3+^ sensing.^76^ B-CDs demonstrated brilliant selectivity and high degree of fluorescence quenching. Furthermore, some works have focused on other acids such as citric acid for improving CDs' performance in biosensing.^77,78^ For instance, Jeong and co-workers,^74^ added oxygen/nitrogen-containing small molecules during the ball-milling process to present fluorescence chemosensor with rapid response behavior based on carboxylic acid-functionalized CDs for Fe^3+^ determination (Fig. 3C). For this purpose, carboxylic acid-functionalized CDs were embedded in a microgel matrix to provide a consistent fluorescence intensity. The designed probe (carboxylic acid-functionalized CDs) revealed high selectivity for detection of Fe^3+^ due to selective sensing behavior under various counter anions. The selectivity and sensitivity of the functionalized CBMs such as CDs and GQDs were improved by using boronic acid and citric acid. There is a massive shortage of both other types of CBMs and acids.

**Fig. 4:**
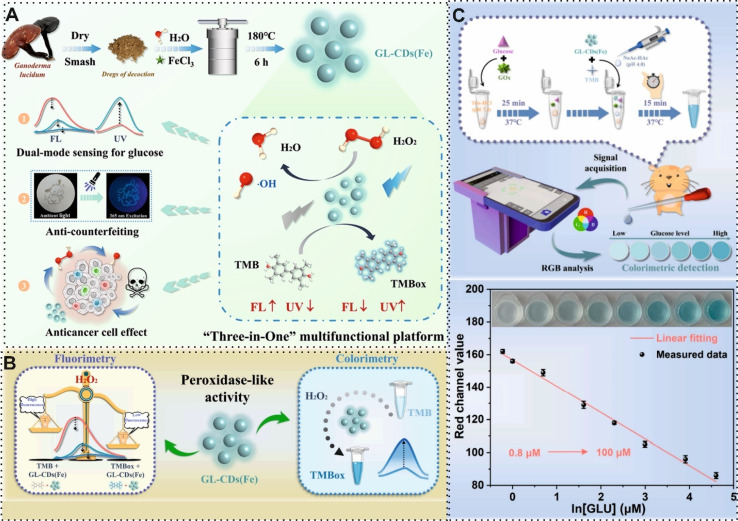
(A) Representation of using CDs, which derived from *Ganoderma lucidum* waste, for introducing “three-in-one” platform and (B and C) dual-mode sensing platform and integration of smartphone for detection of glucose. Reproduced with permission from ref. 80. Copyright Elsevier Science, 2024.

The decoration of BW-derived CBMs with other materials, such as metallic-based, silica-based and magnetic-based materials, is another technique for improving the performance of these CBMs for biomedical application. As an example, Cotchim *et al.*,^79^ prepared a novel and portable EC immunosensor by exploiting the decoration hierarchical microporous carbon material with gold nanoparticles (AuNPs) for ovarian cancer detection. For this purpose, the surface of the screen-printed electrode (SPE) was modified with hierarchical microporous carbon material, which was obtained from waste coffee grounds and, subsequently, the modified surface was decorated with AuNPs. This fabricated surface could present an appropriate immobilization surface for specific antibodies. In the presence of the target, the analysis of EC signals of the antibody–antigen complex was conducted by smartphone-based potentiostat with a LOD of 0.4 U mL^−1^. In another study, Wang and co-workers,^80^ designed a dual-mode sensing approach based on Fe doped into the CDs, which derived from *Ganoderma lucidum* waste, for glucose detection. As shown in Fig. 4A, the *Ganoderma lucidum* waste mixed with Fe during the hydrothermal reaction. Fe-CDs. The presence of Fe in the structure of CDs improved fluorescence signal, catalytic activity, magnetic properties, and stability. Interestingly, Fe-CDs acted as a nanozyme in the reaction between its catalytic intermediate and the chromogenic substrate. This principle was implemented in fluorescence and colorimetric sensors to detect glucose with an excellent LOD of 0.28, and 0.37 μM, respectively. Surprisingly, in this platform, the application of a smartphone could capture the intensity of color and convert the color information into RGB value (Fig. 4B and C). Adding metal oxides in the structure of BW-derived CBMs may result in the agglomeration of the metal oxide nanoparticles. In this regard, instead of removing additional materials, preservation of materials in the structure of these CBMs is a reliable solution for addressing this problem.

**Fig. 5:**
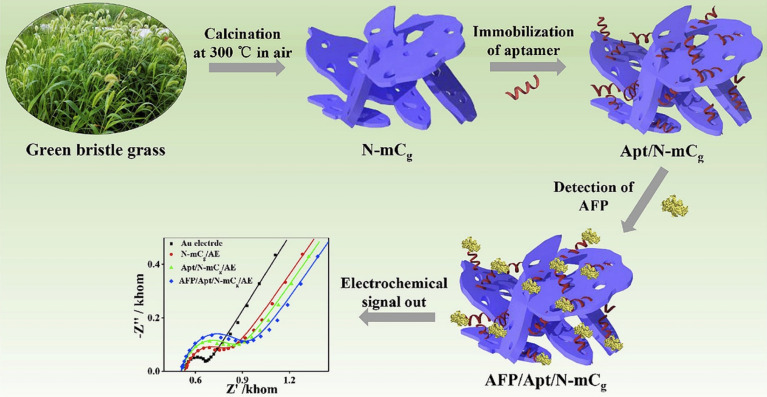
Schematic illustration of EC aptassay which used nanoporous carbon nanomaterials for cancer detection. Reproduced with permission from ref. 82. Copyright Elsevier Science, 2019.

Tea, mushroom, and coffee waste are naturally abundant sources of CBMs from BW for biomedical applications. These BWs have been widely used for synthesis of CBMs which have developed efficient biosensors. The presence of silica materials with CBMs in some BWs, such as rice and sweetcorn husks, has received attention. In many cases, the silica and CBMs of these BWs were separated during transfer process. However, the separation of silica from CBMs is not entirely possible. Therefore, it can be considered a chance for synthesis of conductive and high surface area matrix for immobilization of different bioreceptors. Furthermore, silica materials are dispersed homogenously in the carbon matrix. In 2021, Pandey *et al.*,^81^ prepared a porous carbon with inherent silica derived from sweetcorn husk for introducing a sensitive and selective EC immunosensor of fibronectin protein which is related to ovarian cancer in blood plasma. In this study, the surface of the glassy carbon electrode (GCE) was modified with the synthesized probe for immobilization of antibodies through EDC/NHS cross-linking chemistry. When the target was added to the system, the LOD of 129 fg mL^−1^ was achieved by using EC techniques. The immune response and low stability of antibodies in comparison to aptamer lead to use of aptamer in the structure of biosensor based on BW for biomedical application. In this perspective, Huang and co-workers,^82^ introduced an EC aptasensor based on green bristle grass-derived nitrogen-doped nanoporous carbon nanomaterials for alpha-fetoprotein, as an important biomarker of cancer, detection in real serum samples (Fig. 5). Thanks to the strong affinity for aptamer strands, integration of nitrogen-doped nanoporous carbon nanomaterials with aptamer sequences on the surface of EC electrode revealed excellent the EC activity toward alpha-fetoprotein.

The figure of different analytical methods that exploited the synthesis of carbon frameworks from waste coffee grounds is summarized in Table 3. In detail, the impact of the synthesis method, optimized parameters of the process, and functionalized is undeniable. In favor of the optimization of different parameters, the researchers were able to achieve desirable carbon frameworks which improve the EC and optical signals. Therefore, it can be considered a systematic platform to tailor the waste coffee ground-derived porous carbon. The application of different synthesis methods for activating CBMs in biomedical analysis can introduce high potential methods due to porosity and high surface area. On the other hand, the low catalytic activity demonstrates the necessity of modification of these materials with functionalized groups and metal-based materials. Importantly, there is a massive shortage in the using magnetic materials integrated with CBMs derived from coffee waste owing to their high conductivity. This phenomenon can be achieved by loading magnetic materials into the structure of CBMs derived from coffee waste and constructing a core@shell structure.

**Table 3:** Figures-of-merit of the reported sensors and biosensors based on carbon materials derived from green BWs waste

Detection method	Technique	Synthesis method	Materials	BW	Target	LOD and LLOQ	Linear range	Ref.
EC	Voltammetric	Pyrolysis	Porous carbon	Coffee ground	Neurotransmitter	—	—	62
EC	Voltammetric	Chemical activation	Porous carbon	Amla	Ascorbic acid, dopamine, uric acid, and nitrite	13.7 μM, 3.2 μM, 1.1 μM and 3.3 μM	33 to 166 μM, 1.6 to 72 μM, 1.6 to 4134 and 4.9 to 1184 μM	63
EC	Voltammetric	—	Porous carbon	Shaddock (*Citrus maxima*) peels	Inorganic and organic molecules	3.53 μM	5 to 1760 μM	64
EC	Voltammetric	Chemical activation	Graphene	Rice husk	Mefenamic acid	2.13 nM	1.0 × 10^−8^ to 4.0 × 10^−4^ M	65
Optical	Photoluminescence	Hydrothermal	GQDs	Coffee ground	Glucose	12.45 mM	5 to 45 mM	66
Optical	Fluorescence	Hydrothermal	CDs	Tender coconut	Ethionamide	0.33 μg mL^−1^	0 to 2.4 μg mL^−1^	69
Optical	Fluorescence	Hydrothermal	CDs	Coffee	Fe^3+^ and ascorbic acid	4.314 and 0.162 μM	0 to 100 and 0 to 1 μM	70
Optical	Fluorescence	Hydrothermal	CDs	Seeds of green pepper	Fe^3+^	0.1 μM	1 to 500 μM	71
Optical	Fluorescence	Hydrothermal	CDs	Coffee	Dopamine	4.25 nM	0 to 30 μM	72
Optical	Fluorescence	Hydrothermal	CDs	Coffee	γ-aminobutyric acid	95.09 nM	10 and 20 μM	73
Optical	Fluorescence	Solvothermal	CDs	Chinese herbal residues	Fe^3+^	1.08 μM	0 to 80 μM	76
Optical	Fluorescence	Ball-milling	CDs	Coffee ground	Fe^3+^	2.25 μM	0 to 5.0 mM	74
EC	Voltammetric and impedance	—	Hierarchical microporous carbon	Coffee ground	Ovarian cancer	0.4 U/mL	0.5 to 50.0 U mL^−1^	79
Optical	Fluorescence and colorimetric	Hydrothermal	CDs	*Ganoderma lucidum*	Glucose	0.28 μM	0.8 to 100 μM	80
EC	Voltammetric and impedance	—	Porous carbon	Sweetcorn husk	Ovarian cancer	129 fg mL^−1^	100 fg mL^−1^ to10 μg mL^−1^	81
EC	Voltammetric and impedance	Pyrolysis	Nanoporous carbon nanomaterials	Green bristle grass	Alpha-fetoprotein	60.8 fg mL^−1^	0.1 pg mL^−1^ to 100 ng mL^−1^	82
Optical	Fluorescence and colorimetric	Hydrothermal	CQDs	Coffee grounds	Ascorbic acid	0.133 and 1.56 μM	3.3 to 32.2 μM	83
Optical	Fluorescence	Hydrothermal	CQDs	Coffee grounds	Sodium cyclamate	3.16 μM	2.8 to 56 μM	84
EC	Voltammetric	Hydrothermal	CBMs	Hazelnut shell	Catechol	8.51 nM	0.80 to 80 μM	85
EC	Voltammetric	—	CBMs	Rice halls	Furazolidone and chloramphenicol	0.053 μM and 0.087 μM	0.1 to 1600 μM and 0.1to 3200 μM	86
EC	Voltammetric	Hydrothermal	Activated carbon	Coffee ground	Ciprofloxacin	0.20 nM	0.5 to 25 nM	87
EC	Voltammetric	Pyrolysis	Carbon-based material	Castor cake	Caffeic acid	30.9 nM	1.0 to 3000 μM	88
EC	Voltammetric	Template-assisted	Hierarchically porous carbon	Plant-derived tannin acid	Chlorogenic acid	6.2 nM	0.03 to 1 μM	89
EC	Voltammetric	Pyrolysis	—	Coffee ground	Pb^2+^	4.5 nM	0.128 to 2.44 μM	90
EC	Voltammetric	Hydrothermal	—	Coffee ground	Hydroxychloroquine sulfate and bisphenol A	0.46 μM and 0.31 μM	1.0 to 50 μM and 0.5 to 10 μM	91
Optical	Fluorescence	Hydrothermal	CDs	Flowers of wintersweet	Cr(vi) and Fe^3+^	0.07 μM and 0.15 μM	0.1 to 60 μM and 0.05 to 100 μM	92
Optical	Fluorescence	Hydrothermal	CDs	Tobacco stems	Different tetracycline antibiotics	1.328 nM, 3.234 nM, and 9.881 nM	0.004 to 100.0 μM, 0.011 to 100.0 μM, and 0.033 to 200.0 μM	93
Optical	Fluorescence	Hydrothermal	CDs	Coffee	Pb^2+^ and Cu^2+^ ions	1.358 μg L^−1^ and 0.447 mg L^−1^	0.1 to 50.0 mg L^−1^ and 5.0 to 50 μg L^−1^	94
Optical	Fluorescence	Hydrothermal	CDs	Coffee ground	Noxious nitroanilines	68 ppb	—	95
EC	Voltammetric	Hydrothermal	CDs	Orange peel	Nitrobenzene	13 nM	0.1 to 2000 μM	96
Optical	Fluorescence	—	Carbon materials	Vegetable waste	Alkaline phosphatase	0.25 nM	0.5 to 10 nM	97
Optical	Fluorescence	Hydrothermal	CDs	Coffee beans	Fe^3+^	15.4 mM	0 to 0.10 mM	98
Optical	Fluorescence	—	CDs	Apricot shell	Ascorbic acid	40 nM	1 to 100 μM	99
Optical	Fluorescence	Hydrothermal	Nanodiamond-like carbon	Orange peel	Atropine sulfate	34.42 nM	300 nM to 1 M and from 1 M to 10 M	100
Optical	Fluorescence	Pyrolysis	CDs	Coffee	Picric acid and Fe^3+^	0.26 and 0.83 μM	0 to 0.15 mM	101

### 4.2. Food safety

**Fig. 6:**
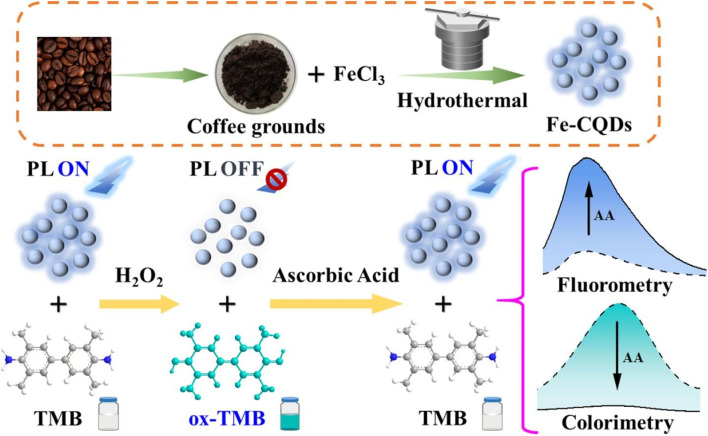
Representation of colorimetric and fluorescence sensor based on magnetic CBMs for ascorbic acid determination. Reproduced with permission from ref. 83. Copyright Elsevier Science, 2023.

Nowadays, due to our busy lifestyle and the high demand for processed foods, our health is more dependent on the quality of food we consume than ever before. The application of BW-derived CBMs in the structure of sensors is a novel and innovative solution for the detection of food contamination in different food matrices. Their biocompatibility, sensitivity, and accessibility offer significant advantages for improving the performance of sensors.^102,103^ Conventionally, BW-derived CBMs were used in gas chromatography-mass spectrometry (GC-MS) for the separation and quantification of complex mixtures of compounds. This method operates according to the integration of two well-established analytical methods including mass spectrometry and gas chromatography.^104–106^ As an example, Song *et al.*,^107^ exploited magnetic carbon particles from coffee grounds in the structure of GC-MS for the analysis of magnetic solid-phase extraction of eight phthalic acid esters in plastic bottled water. However, some limitations and disadvantages can restrict their application in food safety analysis. Indeed, the inability to analyze high molecular weight compounds, limited quantitative accuracy for isomeric compounds, and high initial cost are the most significant limitations. Recently, the application of other types of analytical methods has attracted considerable attention. The analysis of complex food samples by Fe-CQDs, derived from coffee waste, was conducted in colorimetric and fluorescence detection of ascorbic acid in real beverage samples due to strong optical properties.^83^ In this study, fluorescence “on–off–on” mode and peroxidase-like activity were used for fluorescence quenching and converting colorless 3,3′,5,5′-tetramethylbenzidine (TMB) to blue oxTMB, respectively (Fig. 6). Under optimal conditions, the developed dual recognition mechanism enhanced the selectivity and reliability of determination process, introducing a robust sensing platform for monitoring ascorbic acid concentration. Similarly, Chen and colleagues,^84^ reported a simple and sensitive fluorescence turn-on system using CQDs, which were obtained from coffee waste, for sodium cyclamate determination. For this purpose, the difference in fluorescence signal with/without the target was measured by introducing a sensing device with a detection limit of 3.16 μM.

**Fig. 7:**
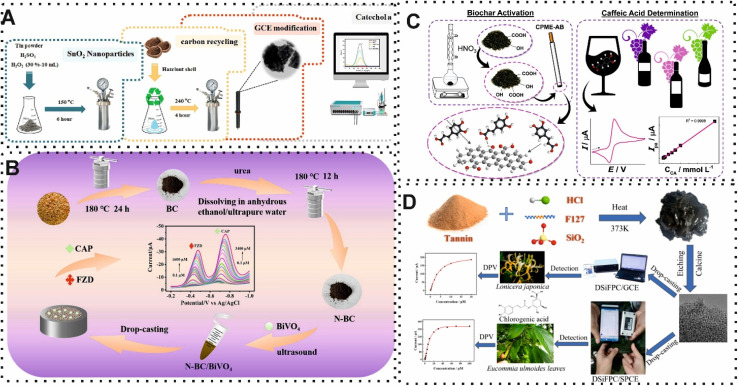
(A) Representation of EC sensor which exploited carbon/SnO_2_ NPs for determination of catechol. Reproduced with permission from ref. 85. Copyright Elsevier Science, 2024. (B) Schematic of using nitrogen-BW derived carbon materials/BiVO_4_ on the surface of GCE for quantification of furazolidone and chloramphenicol. Reproduced with permission from ref. 86. Copyright Elsevier Science, 2024. (C) Illustration of the synthesized castor cake waste-derived CBMs and its application in EC sensor for caffeic acid detection. Reproduced with permission from ref. 88. Copyright Elsevier Science, 2020. (D) Schematic using carbon-based material from plant-derived tannic acid for detection of chlorogenic acid. Reproduced with permission from ref. 89. Copyright Elsevier Science, 2024.

The potential of EC sensors, as highly sensitive and real-time monitoring, in the analyze of food has been highlighted by using BW-derived carbon material as recognition components on the surface of the EC electrode. Interestingly, the performance of these CBMs has been improved by integration of other materials. The electron transfer of redox reactions was facilitated due to their electronic, magnetic, optical and catalytic features.^108^ A variety of materials, including metallic materials (such as AuNPs) and semiconducting materials (like SnO_2_ and BiVO_4_) were used for this purpose. For instance, Gaber and co-workers,^85^ reported a sensitive and selective EC sensor based on the integration of waste hazelnut shells-derived CBMs and SnO_2_ NPs for quantification of catechol in fruit juice and green tea. In this work, the improvement in the performance of platform relied on the electron transfer of synthesized CBMs and the specific surface chemistry of SnO_2_ NPs. In detail, SnO_2_ NPs as a n-type semi-conductivity, demonstrate strong chemical stability and exceptional electrical conductivity. As shown in Fig. 7A, the surface of GCE was modified with carbon/SnO_2_ NPs for capturing catechol and measuring the EC signal with a voltammetric technique. The designed EC sensor revealed an excellent LOD (8.51 nM) and recovery value (102.05%) for green tea and fruit juice. Another EC sensor focused on using BiVO_4_ nanocomposites, as another n-type semiconductor materials, and nitrogen doped in the structure of CBMs from rice hulls for simultaneous determination of furazolidone and chloramphenicol.^86^ The presence of nitrogen in the structure of CBMs changed electron spin distribution and the charge of carbon owing to the polarization of carbon atoms. Furthermore, the exceptional physical and chemical features of BiVO_4_ were exploited for increasing performance. In this regard, the preparation of nitrogen-BW derived carbon materials/BiVO_4_/GCE sensor detected furazolidone and chloramphenicol with a LOD of 0.053 μM and 0.087 μM, respectively (Fig. 7B).

Another promising strategy for improving the performance of EC sensors is surfactants which enhance kinetics of electron transfer reactions and reaction rates. In addition, controlling the solubilization of organic compounds for electroanalysis in water is another advantage of using surfactants. Recently, Gissawong *et al.*,^87^ developed a sensitive and selective EC sensor based on the modification of waste coffee grounds activated carbon with AuNPs for ciprofloxacin quantification in dairy samples. This material introduced an appropriate substrate on the GCE owing to the large surface area, affordability, well-developed pore structure, and chemical stability compared with other alternatives. Indeed, the electron transfer on the surface of EC electrode was significantly improved during the redox reaction of ciprofloxacin. Furthermore, surfactants namely dodecyltrimethylammonium bromide (DTAB) and didodecyldimethylammonium bromide (DDAB) enhanced the EC response. In the presence of the target, the EC signal was measured by a voltammetric technique with a linear range and a LOD of 0.5 to 25 nM and 0.20 nM, respectively. The review of application of carbon materials derived from BW in different methods demonstrated that scholars have attempted to develop compact sensing approaches in food safety. In this regard, EC and fluorescence methods play important roles. However, the exploitation of other optical methods such as surface plasmon resonance (SPR), surface-enhanced Raman scattering (SERS), fluorescence resonance energy transfer (FRET), luminescence, and piezoelectric sensors can be used in the future. Importantly, due to the high potential of EC sensors for miniaturization, application of different analytical approaches with smart and miniaturization gadgets such as smartphones, microfluidics systems, and SPE can present more efficient sensing devices. For example, Kalinke and co-workers,^88^ implemented a low-cost and simple voltammetric sensor on the surface of SPE for detection of caffeic acid. For this purpose, the sensing zone of SPE was modified with synthesized biochar, as a carbon-based material, from castor cake waste for capturing the target. Under normal conditions, the measurement of the voltammetric signal provided a LOD of 30.9 nM (Fig. 7C). The comparison of different reported sensors for food safety is summarized in Table 1. In detail, due to the best results in comparison to other developed sensors, SPE opens many ways for the fabrication of portable sensing approaches. Most recently, Jiang and colleagues,^89^ integrated a smartphone in EC sensor based on hierarchically porous carbon from plant-derived tannin acid for quantification of chlorogenic acid in herbal tea. Several properties of hierarchically porous carbon such as high electrocatalytic activity and large specific surface area introduced an appropriate substrate on the sensing zone of SPE. As shown in Fig. 7D, the modified surface presented the smartphone-operated wireless portable sensor by analyzing the voltammetric signal.

### 4.3. Environmental monitoring

**Fig. 8:**
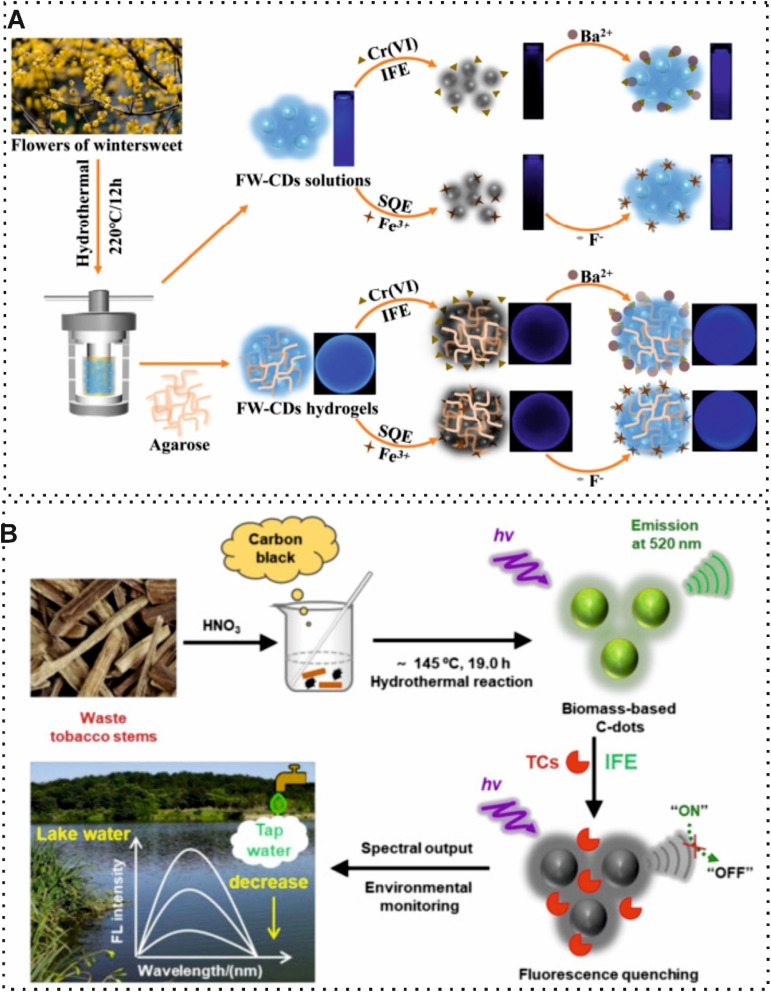
(A) Schematic of fluorescence sensor based on using flowers of wintersweet for synthesizing CDs for visual quantification of Cr(vi) and Fe^3+^.^92^ (B) Illustration of CDs derived from tobacco stems waste for quantification of tetracycline antibiotic residues in water samples.^93^

According to the frequent human activities, the number of harmful substances released into the environment has increased dramatically along with the world's population growth and industrialization. These substances in the water, air, and soil are known in toxicity and may pose health risks. Environmental pollution has become one of the most urgent challenges, capturing the world's attention. The application of BW for introducing affordable and efficient synthesis materials has attracted great attention in environmental analysis.^109,110^ Biochar and hydrochar are two important obtained carbon-rich materials obtained which are used as sensing probes or carbon sources of different carbon nanomaterials. Pyrolysis and hydrothermal carbonization are processes for producing biochar and hydrochar, respectively. Furthermore, there are differences between the properties and applications of biochar and hydrochar. For example, biochar is characterized by its porous structure, stability, and high carbon content. On the other hand, another porous structure may have a higher oxygen content. Recently, some researchers have used these carbon-rich materials in the structure of sensors for environmental monitoring. For instance, Oliveira *et al.*,^90^ developed a useful voltammetric sensor based on biochar obtained from spent coffee grounds for Pb^2+^ detection. In this study, nitric acid (HNO_3_) was used as an oxidizing agent to enhance surface groups. On this account, the activated biochar can interact with inorganic and organic compounds and this phenomenon was considered a principle of detection. Under optimal conditions, the difference of EC signal with/without the presence of the target was measured with a LOD of 4.5 nM which demonstrated that functionalized biochar is promising for sensing since it can adsorb more species than pristine biochar. Most recently, Barreto and co-workers,^91^ exploited a novel analytical approach using spent coffee grounds hydrochar, as a high oxygen content, which was synthesized by hydrothermal carbonization and modified with copper nanoparticles (CuNPs) for hydroxychloroquine sulfate and bisphenol A quantification in natural water. Indeed, the modification of CuNPs in the structure of hydrochar could improve the surface area, conductivity, and stability of the probe due to its excellent electrocatalytic properties. The modification of the EC electrode with hydrochar and CuNPs captures a sensitive and robust EC sensor. In the presence of the target, the EC oxidation of decoration electrode was measured by voltammetric technique with a LOD of 0.46 μM and 0.31 μM for hydroxychloroquine sulfate and bisphenol A, respectively. Along with the application of biochar and hydrochar in sensing, these carbon-rich materials can be used as carbonaceous precursors for the synthesis of other CBMs due to the affordability and ease of process. The conversion of biochar and hydrochar, as large carbon-rich structures, into CDs is carried out through various thermal and chemical treatments.^111^ Instead of this concept, several researchers have attempted to use a directly hydrothermal method for synthesizing CDs. Recently, Xia *et al.*,^92^ developed a straightforward and efficient fluorescence sensor based on the flower waste derived-CDs for visual quantification of Cr(vi) and Fe^3+^ in tap, river, and lake waters (Fig. 8A). In this work, the synthesized CDs through a simple hydrothermal method were used as fluorescent probes for capturing of the targets. The interaction of the developed fluorescence probe with these metal ions changed the intensity of the fluorescence signal. The reported optical sensing device revealed a LOD of 0.07 μM and 0.15 μM for Cr(vi) and Fe^3+^. Similarly, Yang and co-workers,^93^ utilized waste tobacco stems to synthesize novel CDs for fluorescence detection of tetracycline antibiotic residues in water samples. The conversion of waste tobacco stems into CDs, as a fluorescence probe, was conducted by a one-pot hydrothermal method which operated without multiple purification or processing steps. The detection principle of the reported sensing approach relied on the fluorescence response of CDs toward different tetracycline antibiotics (Fig. 8B).

In order to improve the refinement and provide highly functionalized or specialized CDs, Christopher and co-workers,^94^ fabricated an efficient and simple fluorescence-based sensor, which used a multi-step approach consisting of tandem hydrothermal and pyrolysis process, for detection of Pb^2+^ and Cu^2+^ ions. In detail, the fluorescence of CDs was directly quenched by these ions linearly. Hence, the fluorescence intensity decreased by increasing the concentration of the target. In 2022, CDs derived from spent coffee grounds by integration of hydrothermal carbonization and microwave-assisted hydrothermal carbonization optimized synthesis conditions and improved the properties of CDs for detection of noxious nitroanilines.^95^ For this purpose, microwave-assisted hydrothermal carbonization and hydrothermal carbonization techniques were used for synthesizing desirable CDs with quantum yields reaching up to 0.18, which can be considered excellent in terms of fluorescent properties. In the presence of *para*-nitroaniline, the quenching of fluorescence indicated a LOD of 68 ppb. In another study, Bressi and co-workers,^96^ combination of hydrothermal carbonization and EC to synthesize CDs from waste orange peels for voltammetric detection of nitrobenzene. For this purpose, the obtained liquid phase of hydrothermal carbonization was used as a precursor for EC synthesis. Due to the excellent EC properties of CDs, the sensing zone of the SPE was modified with CDs for capturing nitrobenzene in water. Under normal conditions, the prepared EC sensor detected nitrobenzene by measuring the voltammetric technique.

All in all, although biochar and hydrochar have attracted considerable attention in sensing, there are few investigations on their usage in EC and optical sensing. Hence, it is expected that the number of probes based on these materials derived from BW will be increased. Furthermore, these materials have an important role in synthesis CDs which are widely used in optical sensors. Therefore, the studies of both of these can broaden researchers' horizons about the application of coffee waste as a source of materials in sensing.

## 5. Conclusion and future perspective

BW has attracted considerable attention in both green chemistry and analytical chemistry for numerous applications due to their potential as valuable precursors of CBMs and nanomaterials. The conversion of these BWs to CBMs is considered one of the excellent instances of “waste to wealth”. The importance of producing herbaceous and animal waste is undervalued, however, new emerging studies demonstrate the high value of these wastes which can be recovered due to nutritional and economic value. Indeed, different synthesis methods based on the use of BWs for producing materials, which are used in sensing, are considerably attractive due to high-valued materials that can be obtained from low-value precursors, leading to a circular economy. Over the last decades, researchers have used different synthesis methods such as hydrothermal carbonization, ball-milling, pyrolysis, and lonothermal carbonization techniques for addressing environmental concerns associated with BW disposal and introducing numerous CBMs which have been utilized in the structure of various analytical methods for biomedical application, food safety, and environmental monitoring.

The potential of CBMs obtained from BW can be analyzed in various fields. Despite the rapid progress made in synthesizing CBMs, developing a production system that satisfies both environmental and industrial requirements is a challenging task. The system must ensure scalability, quality control, and safety while remaining non-hazardous and simple. The use of non-toxic reagents is a top priority. Nontoxic reagents can produce safe materials with many advantages in terms of environmental and health concerns. Another important matter is to minimize carbon emissions by introducing an energy-efficient synthetic process. Therefore, discovering the ideal waste coffee and developing eco-friendly methods for synthesizing CBMs has been a long-awaited goal. Although there is currently no widespread use of BW, these wastes can be considered a relatively unique type of organic waste. In this regard, the activation of carbon precursors by activators remains challenging, synthesizing uncontrollable pore distribution and low carbon yield. In addition, it is important to find accessible and affordable sources of carbon precursors. These wastes and techniques should be optimized for achieving a high yield of waste-derived CBMs.

## Data availability

No data were used for the research described in the article.

## Conflicts of interest

The authors declare no conflict of interest.

## References

[cit1] Ganivet E. (2020). Environ. Dev. Sustain..

[cit2] Sharma P., Gaur V. K., Kim S.-H., Pandey A. (2020). Bioresour. Technol..

[cit3] Ling R. L. Z., Kong L. K., Lim L. H., Teo S. S., Ng H.-S., Lan J. C.-W., Khoo K. S. (2023). Environ. Res..

[cit4] Adebayo F., Obiekezie S. (2018). Res. J. Sci. Technol..

[cit5] Yousaf B., Liu G., Abbas Q., Wang R., Imtiaz M., Zia-ur-Rehman M. (2017). Land Degrad. Dev..

[cit6] Zhi L., Zhipeng R., Minglong L., Rongjun B., Xiaoyu L., Haifei L., Kun C., Xuhui Z., Jufeng Z., Lianqing L. (2020). J. Clean. Prod..

[cit7] Vilas-Boas A. A., Pintado M., Oliveira A. L. (2021). Foods.

[cit8] Pham X. N., Vu V.-T., Nguyen H. V. T., Nguyen T.-T.-B., Doan H. V. (2022). Nanoscale Adv..

[cit9] Van D. P., Fujiwara T., Tho B. L., Toan P. P. S., Minh G. H. (2020). Environ. Res. Eng. Manag..

[cit10] Wang Q., Tian D., Hu J., Shen F., Yang G., Zhang Y., Deng S., Zhang J., Zeng Y., Hu Y. (2018). RSC Adv..

[cit11] Prismantoko A., Karuana F., Nugroho A., Santoso P. A., Putra H. P., Darmawan A., Muflikhun M. A., Pranoto I., Aziz M., Hariana H. (2024). Waste Manage..

[cit12] Van der Sloot H., Kosson D., Van Zomeren A. (2017). Waste Manage..

[cit13] Tursi A. (2019). Biofuel Res. J..

[cit14] Tajik S., Dourandish Z., Zhang K., Beitollahi H., Van Le Q., Jang H. W., Shokouhimehr M. (2020). RSC Adv..

[cit15] Kumar Y. R., Deshmukh K., Sadasivuni K. K., Pasha S. K. (2020). RSC Adv..

[cit16] Ahmad H., Khan R. A., Koo B. H., Alsalme A. (2022). RSC Adv..

[cit17] Kordasht H. K., Hasanzadeh M. (2020). J. Mol. Recogn..

[cit18] Kordasht H. K., Pazhuhi M., Pashazadeh-Panahi P., Hasanzadeh M., Shadjou N. (2020). TrAC, Trends Anal. Chem..

[cit19] Kordasht H. K., Hasanzadeh M., Seidi F., Alizadeh P. M. (2021). TrAC, Trends Anal. Chem..

[cit20] Bosu S., Rajamohan N., Sagadevan S., Raut N. (2023). Chemosphere.

[cit21] Malode S. J., Shanbhag M. M., Kumari R., Dkhar D. S., Chandra P., Shetti N. P. (2023). J. Pharm. Biomed. Anal..

[cit22] Blessy Rebecca P., Durgalakshmi D., Balakumar S., Rakkesh R. A. (2022). ChemistrySelect.

[cit23] Katinas V., Marčiukaitis M., Perednis E., Dzenajavičienė E. F. (2019). Renew. Sustain. Energy Rev..

[cit24] Liu X., Sun Y., Tang Y., Wang M., Xiao B. (2023). Chemosphere.

[cit25] Roni M. S. , Hartley D. S., Griffel M., Hu H., Nguyen Q. A., Cai H. and Thompson D. N., Herbaceous Feedstock 2018 State of Technology Report, Idaho National Lab.(INL), Idaho Falls, ID (United States), 2020

[cit26] Kanageswari S. V., Tabil L. G., Sokhansanj S. (2022). Energies.

[cit27] Kang B.-J., Jeon J.-M., Bhatia S. K., Kim D.-H., Yang Y.-H., Jung S., Yoon J.-J. (2023). Polymers.

[cit28] Yang B., Shao X., Gu X., Wang K., Ning X., Xia J., Xie M., Tang Y., Li Q., Tian S. (2023). Chem. Eng. J..

[cit29] Klein H. S. and Luna F. V., Brazilian Crops in the Global Market: the Emergence of Brazil as a World Agribusiness Exporter since 1950, Springer Nature, 2023

[cit30] Forcina A., Petrillo A., Travaglioni M., di Chiara S., De Felice F. (2023). J. Clean. Prod..

[cit31] Zahniser S. , Johnson W. and Valdes C., Changes in US Agricultural Imports From Latin America and the Caribbean, ERS-AES-124, https://www.ers.usda.gov/webdocs/outlooks/106972/aes-124-01.pdf, 2023

[cit32] Lee Y.-G., Cho E.-J., Maskey S., Nguyen D.-T., Bae H.-J. (2023). Molecules.

[cit33] Mak S. L., Wu M. Y. T., Chak W. Y., Kwong W. K., Tang W. F., Li C. H., Lee C. C., Li C. Y. (2023). Sustainability.

[cit34] Madu I. E., Kamaruddin M. A., Yusoff M. S., Niza N. M., Shadi A. M. H., Norashiddin F. A. (2022). Coffee Sci..

[cit35] El-Sayed I. M., Salama W. H., Badr M. (2024). Plant Physiol. Biochem..

[cit36] Shin J., Kwak J., Kim S., Son C., Lee Y.-G., Kim J., Bae S., Park Y., Lee S.-H., Chon K. (2023). Chem. Eng. J..

[cit37] Samoraj M., Mironiuk M., Izydorczyk G., Witek-Krowiak A., Szopa D., Moustakas K., Chojnacka K. (2022). Chemosphere.

[cit38] Checcucci A., Trevisi P., Luise D., Modesto M., Blasioli S., Braschi I., Mattarelli P. (2020). Front. Microbiol..

[cit39] Simsek S., Uslu S., Simsek H. (2022). Energy.

[cit40] Shurson G. C. (2020). Sustainability.

[cit41] Kumar R., Singh R. K., Singh D. P. (2016). Renew. Sustain. Energy Rev..

[cit42] Colmenares J. C., Varma R. S., Lisowski P. (2016). Green Chem..

[cit43] Budsaereechai S., Hunt A. J., Ngernyen Y. (2019). RSC Adv..

[cit44] Wang L., Lei H., Liu J., Bu Q. (2018). RSC Adv..

[cit45] Santana M. S., Alves R. P., Santana L. S., Gonçalves M. A., Guerreiro M. C. (2022). J. Environ. Manag..

[cit46] Luz F. C., Volpe M., Fiori L., Manni A., Cordiner S., Mulone V., Rocco V. (2018). Bioresour. Technol..

[cit47] Baruah P., Das B. K., Bora M., Saikia B. K., Mahanta D. (2022). Mater. Today Commun..

[cit48] Yang H., Zhou J., Bao J., Ma Y., Zhou J., Shen C., Luo H., Yang M., Hou C., Huo D. (2021). Microchem. J..

[cit49] Meng A., Huangfu B., Sheng L., Hong X., Li Z. (2022). Microchem. J..

[cit50] Lin X., Xiong M., Zhang J., He C., Ma X., Zhang H., Kuang Y., Yang M., Huang Q. (2021). Microchem. J..

[cit51] Li H.-C., Ji X.-Y., Pan X.-Q., Liu C., Liu W.-J. (2020). ACS ES&T Eng..

[cit52] Ayoub M., Alami A. H., Abdelkareem M. A., Olabi A. (2024). Appl. Surf. Sci. Adv..

[cit53] Xiao L., Zhang Y., Wang X., Hao G., Liu J., Ke X., Chen T., Jiang W. (2018). RSC Adv..

[cit54] Wang G., Jia J., He Y., Wei D., Song M., Zhang L., Li G., Li H., Yuan B. (2022). RSC Adv..

[cit55] Dai L., Zhou N., Lv Y., Cheng Y., Wang Y., Liu Y., Cobb K., Chen P., Lei H., Ruan R. (2022). Prog. Energy Combust. Sci..

[cit56] González-Arias J., Sánchez M. E., Cara-Jiménez J., Baena-Moreno F. M., Zhang Z. (2022). Environ. Chem. Lett..

[cit57] Cibien L., Parot M., Fotsing P. N., Gaveau P., Woumfo E. D., Vieillard J., Napoli A., Brun N. (2020). Green Chem..

[cit58] Bangar S. P., Singh A., Ashogbon A. O., Bobade H. (2023). Int. J. Biol. Macromol..

[cit59] Pavlenko V., Żółtowska S., Haruna A., Zahid M., Mansurov Z., Supiyeva Z., Galal A., Ozoemena K., Abbas Q., Jesionowski T. (2022). Mater. Sci. Eng. R Rep..

[cit60] Ehtesabi H. (2020). Mater. Today Chem..

[cit61] Silva L. R., Carvalho J. H., Stefano J. S., Oliveira G. G., Prakash J., Janegitz B. C. (2023). Mater. Today Commun..

[cit62] Ostertag B. J., Syeed A. J., Brooke A. K., Lapsley K. D., Porshinsky E. J., Ross A. E. (2024). ACS Sens..

[cit63] Sudha V., Kumar S. M. S., Thangamuthu R. (2019). Colloids Surf., B.

[cit64] Sha T., Li X., Liu J., Sun M., Wang N., Bo X., Guo Y., Hu Z., Zhou M. (2018). Sens. Actuators, B.

[cit65] Malode S. J., Joshi M., Shetti N. P., Alshehri M. A. (2024). Mater. Today Commun..

[cit66] Hadish F., Chiang M.-H., Hsieh Y.-F., Wu S.-Y., Jou S. (2022). Adv. Mater. Sci. Eng..

[cit67] Kaur G., Rani R., Raina J., Singh I. (2024). Crit. Rev. Anal. Chem..

[cit68] Wareing T., Phan A., Gentile P., Cucinotta F. (2023). J. Tech. Educ..

[cit69] Gunjal D. B., Gore A. H., Bhosale A. R., Naik V. M., Anbhule P. V., Shejwal R. V., Kolekar G. B. (2019). J. Photochem. Photobiol., A.

[cit70] Won S., Kim J. (2022). Korean J. Chem. Eng..

[cit71] Wang W., Chen J., Wang D., Shen Y., Yang L., Zhang T., Ge J. (2021). Anal. Methods.

[cit72] Sangubotla R., Won S., Kim J. (2023). J. Photochem. Photobiol., A.

[cit73] Sangubotla R., Kim J. (2023). Ceram. Int..

[cit74] Jeong G., Park C. H., Yi D., Yang H. (2023). J. Clean. Prod..

[cit75] Ding Q., Guo Z., Chen W., Yu H., Zhu X., Liu Q., Fu M. (2021). J. Colloid Interface Sci..

[cit76] Zhang L., Luo W., Chen Y., Zheng J., Cao L., Duan L., Tang T., Wang Y. (2023). J. Clean. Prod..

[cit77] Martín-Pacheco A., Del Río Castillo A. E., Martín C., Herrero M. a. A., Merino S., Garcia Fierro J. L., Díez-Barra E., Vázquez E. (2018). ACS Appl. Mater. Interfaces.

[cit78] Youh M.-J., Jiang M.-Y., Chung M.-C., Tai H.-C., Li Y.-Y. (2020). Inorg. Chem. Commun..

[cit79] Cotchim S., Thavarungkul P., Kanatharana P., Thantipwan T., Jiraseree-Amornkun A., Wannapob R., Limbut W. (2023). Microchim. Acta.

[cit80] Wang L., Zheng S., Liu Y., Ji Y., Liu X., Wang F., Li C. (2024). Talanta.

[cit81] Pandey U., Rani M. U., Deshpande A. S., Singh S. G., Agrawal A. (2021). Electrochim. Acta.

[cit82] Huang X., Cui B., Ma Y., Yan X., Xia L., Zhou N., Wang M., He L., Zhang Z. (2019). Anal. Chim. Acta.

[cit83] Zhu Y., Deng X., Chen J., Hu Z., Wu F. (2023). Food Chem..

[cit84] Chen J., Du H., Xu Y., Ma B., Zheng Z., Li P., Jiang Y. (2021). J. Mater. Sci.: Mater. Electron..

[cit85] Gaber A., Bilge S., Donar Y. O., Sınağ A. (2024). Microchem. J..

[cit86] Wang M., Wang Q., Wang X., Wang S. (2024). Microchem. J..

[cit87] Gissawong N., Srijaranai S., Boonchiangma S., Uppachai P., Seehamart K., Jantrasee S., Moore E., Mukdasai S. (2021). Microchim. Acta.

[cit88] Kalinke C., Zanicoski-Moscardi A. P., de Oliveira P. R., Mangrich A. S., Marcolino-Junior L. H., Bergamini M. F. (2020). Microchem. J..

[cit89] Jiang W., Zhuo Z., Zhang X., Luo H., He L., Yang Y., Wen Y., Huang Z., Wang P. (2024). Food Chem..

[cit90] Oliveira G. A., Gevaerd A., Mangrich A. S., Marcolino-Junior L. H., Bergamini M. F. (2021). Microchem. J..

[cit91] Barreto F. C., Ito E. Y., Mounienguet N. K., Dal'Evedove Soares L., Yang J., He Q., Cesarino I. (2023). Chemosensors.

[cit92] Xia L., Li X., Zhang Y., Zhou K., Yuan L., Shi R., Zhang K., Fu Q. (2022). Molecules.

[cit93] Yang H., Wei Y., Yan X., Nie C., Sun Z., Hao L., Su X. (2022). Nanomaterials.

[cit94] Christopher K., Mas’ud Z. A., Hanif N. (2019). Int. J. Eng. Res. Sci. Technol..

[cit95] Costa A. I., Barata P. D., Moraes B., Prata J. V. (2022). Chemosensors.

[cit96] Bressi V., Chiarotto I., Ferlazzo A., Celesti C., Michenzi C., Len T., Iannazzo D., Neri G., Espro C. (2023). Chemelectrochem.

[cit97] Kim M. I., Park S. Y., Park K. S., Kim S.-r., Kim J.-P., Lee Y.-C., Lee H. U., Park H. G. (2018). Sens. Actuators, B.

[cit98] Zhang W., Jia L., Guo X., Yang R., Zhang Y., Zhao Z. (2019). Analyst.

[cit99] Hong-Bo X., Sheng-Hai Z., Ming-Yue L., Zhang P.-R., Zi-Han W., Yan-Mei T., Xu-Qin W. (2022). Chin. J. Anal. Chem..

[cit100] Ramya A. V., Balachandran M. (2021). Front. Environ. Sci. Eng..

[cit101] Zong S., Wang B., Ma W., Yan Y., Li J. (2021). Chem. Res. Chin. Univ..

[cit102] Kishor R., Purchase D., Saratale G. D., Saratale R. G., Ferreira L. F. R., Bilal M., Chandra R., Bharagava R. N. (2021). J. Environ. Chem. Eng..

[cit103] Zazouli M. A., Ghanbari F., Yousefi M., Madihi-Bidgoli S. (2017). J. Environ. Chem. Eng..

[cit104] Beale D. J., Pinu F. R., Kouremenos K. A., Poojary M. M., Narayana V. K., Boughton B. A., Kanojia K., Dayalan S., Jones O. A., Dias D. A. (2018). Metabolomics.

[cit105] Al-Rubaye A. F., Hameed I. H., Kadhim M. J. (2017). Int. J. Toxicol. Pharmacol. Res..

[cit106] Giussani B., Gorla G., Riu J. (2024). Crit. Rev. Anal. Chem..

[cit107] Song N. E., Lim M. C., Choi S. W., Kim D. O., Nam T. G. (2020). J. Food Sci..

[cit108] Nakhlband A., Kholafazad-Kordasht H., Rahimi M., Mokhtarzadeh A., Soleymani J. (2022). Microchem. J..

[cit109] Liu B., Zhuang J., Wei G. (2020). Environ. Sci.: Nano.

[cit110] Martínez I. V., Ek J. I., Ahn E. C., Sustaita A. O. (2022). RSC Adv..

[cit111] Xu M., Kang Y., Jiang L., Jiang L., Tremblay P.-L., Zhang T. (2022). Int. J. Hydrogen Energy.

